# Vibro-Acoustic Platelet Activation: An Additive Mechanism of Prothrombosis with Applicability to Snoring and Obstructive Sleep Apnea

**DOI:** 10.3390/bioengineering10121414

**Published:** 2023-12-12

**Authors:** Daniel E. Palomares, Phat L. Tran, Catherine Jerman, Moe Momayez, Pierre Deymier, Jawaad Sheriff, Danny Bluestein, Sairam Parthasarathy, Marvin J. Slepian

**Affiliations:** 1 Department of Biomedical Engineering, University of Arizona, Tucson, AZ 85724, USA; dpalomares@arizona.edu; 2 Arizona Center for Accelerated Biomedical Innovation, University of Arizona, Tucson, AZ 85724, USA; lephat.tran@gmail.com (P.L.T.); mmomayez@arizona.edu (M.M.); deymier@arizona.edu (P.D.); spartha1@arizona.edu (S.P.); 3 Department of Medicine, University of Arizona, Tucson, AZ 85724, USA; cfj@arizona.edu; 4 Department of Mining & Geological Engineering, University of Arizona, Tucson, AZ 85724, USA; 5 Department of Materials Science & Engineering, University of Arizona, Tucson, AZ 85724, USA; 6 Department of Biomedical Engineering, Stony Brook University, Stony Brook, NY 11794, USA; jawaad.sheriff@stonybrook.edu (J.S.); danny.bluestein@stonybrook.edu (D.B.); 7 Health Sciences Center for Sleep and Circadian Sciences, University of Arizona, Tucson, AZ 85724, USA

**Keywords:** platelet activation, snoring, vibration, obstructive sleep apnea, fluid–structure interactions, shear stress, aspirin

## Abstract

**Introduction:** Obstructive sleep apnea (OSA) and loud snoring are conditions with increased cardiovascular risk and notably an association with stroke. Central in stroke are thrombosis and thromboembolism, all related to and initiaing with platelet activation. Platelet activation in OSA has been felt to be driven by biochemical and inflammatory means, including intermittent catecholamine exposure and transient hypoxia. We hypothesized that snore-associated acoustic vibration (SAAV) is an activator of platelets that synergizes with catecholamines and hypoxia to further amplify platelet activation. **Methods:** Gel-filtered human platelets were exposed to snoring utilizing a designed vibro-acoustic exposure device, varying the time and intensity of exposure and frequency content. Platelet activation was assessed via thrombin generation using the Platelet Activity State assay and scanning electron microscopy. Comparative activation induced by epinephrine and hypoxia were assessed individually as well as additively with SAAV, as well as the inhibitory effect of aspirin. **Results:** We demonstrate that snore-associated acoustic vibration is an independent activator of platelets, which is dependent upon the dose of exposure, i.e., intensity x time. In snoring, acoustic vibrations associated with low-frequency sound content (200 Hz) are more activating than those associated with high frequencies (900 Hz) (53.05% vs. 22.08%, *p* = 0.001). Furthermore, SAAV is additive to both catecholamines and hypoxia-mediated activation, inducing synergistic activation. Finally, aspirin, a known inhibitor of platelet activation, has no significant effect in limiting SAAV platelet activation. **Conclusion:** Snore-associated acoustic vibration is a mechanical means of platelet activation, which may drive prothrombosis and thrombotic risk clinically observed in loud snoring and OSA.

## 1. Introduction

Obstructive sleep apnea (OSA) is an increasingly prevalent, clinically significant condition affecting 2% to 14% of community screened populations with an associated heavy economic burden [1,2,3,4]. Sleep-related changes in muscle tone in OSA lead to intermittent upper airway collapse, increased airway resistance, breathing pauses, and recurrent episodes of hypoxia and hypercapnia during sleep [2,5,6,7,8,9,10,11,12,13]. These reductions (hypopneas) and cessations (apneas) of inspiratory flow provoke arousal from sleep with excitation of the sympathetic nervous system with catecholamine release [2,5,7,8,10,11,13,14,15,16]. Although loud snoring, early-morning headache, and daytime sleepiness are common presenting complaints, the significance of OSA relates to the association and causal relationship to a wide range of disease states burdened with significant morbidity and mortality. In particular, OSA is associated with atrial fibrillation, congestive heart failure, hypertension, coronary artery disease, diabetes mellitus, and stroke [2,3,9,13,14,15,17].

One of the most devastating consequences of OSA is stroke. OSA significantly increases the risk of stroke independent of other cardiovascular and cerebrovascular risk factors, including hypertension [2,5,8,11,12,15,17,18]. Central in OSA stroke is thrombosis. OSA-mediated stroke is largely ischemic, caused by underlying intra-arterial thrombosis or thromboemboli related to an associated prothrombotic state [8,9,11,13,15,18,19,20]. Prothrombosis in OSA is thought to be driven by sympathetic activation, catecholamine surge, and acute blood pressure changes during apneic episodes, resulting in oxidative stress, endothelial dysfunction, and inflammation [10,14,18,21,22,23,24]. Additionally, platelet dysfunction, including increased spontaneous activation and aggregation in patients with OSA, has been previously reported [11,15,19,21,25,26,27,28,29,30]. Hypoxia is known to facilitate prothrombosis via enhanced platelet activation, aggregation, and adhesion and may play a role in the association of OSA and stroke [21,26,28,29,30,31]. Despite these described biochemical and cellular mechanisms, our current understanding of the mechanisms of hypercoagulability and platelet dysfunction in OSA remains incomplete.

Physical forces such as shear stress and pressure are known drivers of platelet activation independent of biochemical agonists [28,29,30]. The cell membrane serves as the first point of contact between a cell and its environment, sensing external cues and transducing these inputs internally, resulting in a phenotypic response. In addition, cells possess a wide range of mechanoreceptors that transduce and transmit extracellular physical force intracellularly via the process of mechanotransduction [32,33,34]. Vibration is an additional physical force that may be transduced by cells [35]. Vibration of anatomic structures in the pharyngeal airway causes snoring [5,36,37,38]. OSA is strongly associated with snoring, specifically loud, vibratory snoring in the low-frequency range [39,40]. Snore-mediated vibrations, over a range of acoustic frequencies, may be transmitted through retropharyngeal tissues, including the carotids and other vasculature, representing a potential source of external physical force sufficient to activate platelets independent of other triggers of platelet activation such as hypoxia or sympathetic activation [36,37,41,42,43]. Via this transmission, fluid–structure interactions further transmit forces to flowing blood and contain platelets moving through both the carotid artery and jugular veins, which may affect platelet activation [5,16,19,44]. We explore this potential here.

In the present study, we hypothesized that snore-associated acoustic vibration (SAAV) is an activator of platelets that will synergize with catecholamines and hypoxia to further amplify platelet activation. To test this hypothesis, we first exposed platelets to snore-associated acoustic vibration using a designed vibro-acoustic exposure device, examining platelet activation and the dose dependency of snore vibration as an activator. As a second step, we explored the synergistic effects of hypoxia and/or exogenous epinephrine with SAAV on platelet activation. Finally, to limit SAAV-mediated platelet activation, we examined the effect of exogenous aspirin on limiting snore-mediated platelet activation.

## 2. Methods

### 2.1. Fabrication of Vibro-Acoustic Exposure Device

**Figure 1:**
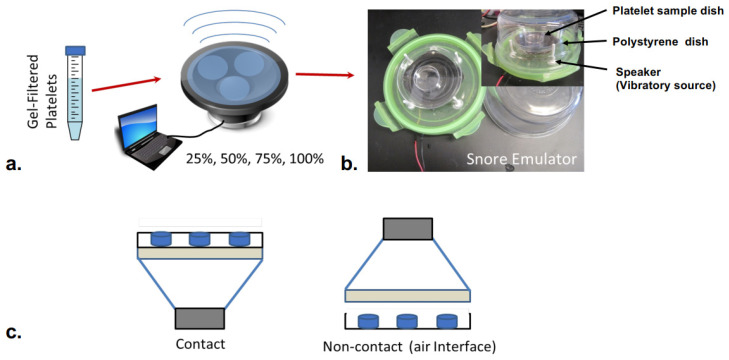
Vibro-Acoustic Exposure Device (VAED). (**a**) Gel-filtered platelets were exposed to snoring in either a contact or non-contact configuration. Snore intensity was varied via computer-controlled digital input over a range (25–100%). (**b**) In the contact mode, the large Petri dish holding samples is in intimate contact with and supported by the speaker membrane. In contact mode, samples are exposed to both sound waves emitted by the speaker as well as vibrations associated with the motion of the speaker membrane. (**c**) In non-contact mode, samples are only subjected to acoustic waves propagating from the speaker toward the Petri dish through an air interface.

A vibro-acoustic exposure device (VAED) was fabricated to allow in vitro exposure of platelets to snoring recorded from human volunteers in a sleep lab or to other sounds/tones. Two configurations of the VAED were designed for differing vibro-acoustic exposure: contact and non-contact (Figure 1). The contact design was formed using a midrange speaker, Petri dishes, and securing tape. A commercial midrange speaker, 13.35 cm in diameter (J.W. Speakers, Germantown, WI, USA), with emitting frequencies between 100 and 8000 Hz was selected. A crystal polystyrene Petri dish (145 mm, Greiner Bio-One, Kremsmünster, Austria) was mounted on the midrange speaker using electrical tape (Scotch, 3M, Vinyl). The polystyrene dish was firmly secured in intimate contact across the face of the speaker, allowing transmitted sound to induce vibration in the affixed Petri dish platform. Smaller 35 mm diameter Petri dishes (Greiner Bio-One, Kremsmünster, Austria) containing desired platelet samples were then similarly taped onto the large Petri dish surface to perform an experiment. In the non-contact design, the speaker was suspended in a frame 1 cm above the 145 mm diameter Petri dish, which was secured to a firm base. Similar to the contact design, smaller 35 mm diameter Petri dishes (Greiner Bio-One, Kremsmünster, Austria) containing desired platelet samples were then identically affixed within the large Petri dish surface to perform an experiment.

For experiments, the VAED was actuated via a digital input signal derived from the snore recording—either as a composite of frequencies (whole snore) or a defined narrow bandwidth of frequency contained within snoring (200 Hz. and 900 Hz.). To measure vibration induced by the VAED, a laser vibrometer (VibroOne, Polytec GmbH) was utilized to obtain the amplitude of the vibro-acoustic wave emitted at different levels of amplification.

### 2.2. Platelet Preparation

Whole blood was collected from consenting healthy adult volunteers of both sexes who had not taken aspirin or ibuprofen for two weeks in accordance with a University of Arizona IRB-approved protocol (protocol #1810013264A002). Whole blood was centrifuged to obtain platelet-rich plasma (PRP), which was filtered through a column of Sepharose 2B beads (Sigma-Aldrich, St. Louis, MO, USA) to collect gel-filtered platelets (GFP) [45]. GFP was diluted to a count of 20,000/μL in HEPES-modified Tyrode’s buffer, with 3 mM CaCl_2_ added 10 min prior to experiments.

### 2.3. Platelet Activation State

The platelet activation state (PAS) is a well-characterized means of measuring mechanically mediated platelet activation and was utilized for all studies herein [46,47]. The PAS assay records the rate of thrombin generation, utilizing acetylated prothrombin while incubating with factor Xa over 10 min at 37 °C at a final platelet count of 5000/µL. To ensure linear kinetics, the feedback action of generated thrombin is blocked via the use of acetylated prothrombin [47]. For all experiments, PAS values were normalized to a sonicated sample of platelets using Equation (1). For sonication, platelet samples were activated using a Branson Sonifier 150, at 10 W for 10 s with a microprobe (Branson, Branson, MO, USA). The sonicated platelets act as a positive control for mechanically activating a platelet. For all protocols outlined herein, serial 25 µL aliquots of GFP samples were analyzed via this assay.

(1) $$Normalized\;PAS=\frac{Experimental\;PAS}{Sonicated\;PAS}$$

### 2.4. Effect of Snoring on Platelet Activation

Gel-filtered platelets were added to the smaller sample Petri dishes and were then exposed to a snore recording (whole snore composite) for 40 min via the VAED, with unexposed GFP in Petri dishes serving as control. All samples were incubated at 37 °C. At serial time points of 0, 10, 20, 30, and 40 min, serial GFP aliquots were collected from both vibration and non-vibration groups, and platelet activation was assessed via the PAS assay.

### 2.5. Scanning Electron Microscopy

Scanning electron microscopy (SEM) was used to visualize morphological changes in platelets exposed to snore vibration. GFP (20,000 platelets/µL) was exposed to snore vibration using the VAED as above, with non-vibration controls, and samples were collected at times 0, 20, and 40 min. Aliquots, 30 μL, of each collected sample were placed on glass coverslips (13 mm diameter) and immediately fixed with the addition of 30 µL of 2% *v*/*v* glutaraldehyde in HEPES-modified platelet buffer for 30 min. Coverslips were rinsed with sequential dilutions of glutaraldehyde in ddH_2_O, then dehydrated with a series of increasing ethanol concentrations reaching 100%. Samples were gold-coated using an Anatech Hummer 6.6 (Anatech, Sparks, NV, USA) sputter system. SEM images were acquired with a FEI Inspec-S SEM (FEI Company, Hillsboro, OR, USA) at 1–10,000×.

### 2.6. Effect of Vibro-Acoustic Stimulation on Platelet Activation

**Figure 2:**
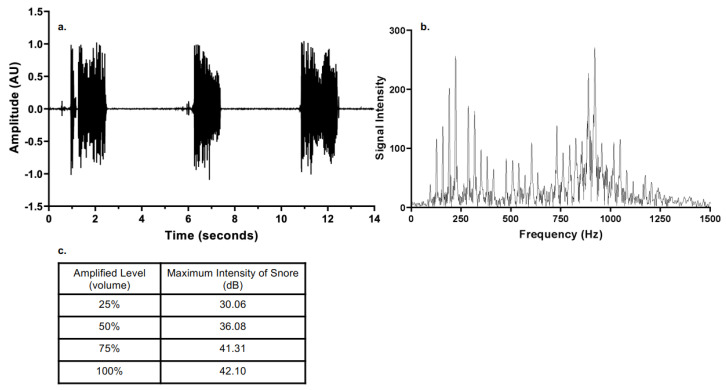
(**a**) Typical OSA snore-note periodic active and silent intervals. (**b**) Frequency spectrum of snore. (**c**) Maximum intensity of snore as a function of amplified level.

For frequency band-specific studies, 200 Hz and 900 Hz were selected as the exposure frequencies based on the identified peak frequency regions (Figure 2b) of recorded snoring. The open-source software Audacity^®^ (version 2.2.2) generated each frequency as a continuous sinusoidal wave for 40 min using an amplitude of 1AU (Arbitrary Units), and this was utilized for activation of the VAED. Each frequency was tested individually to determine its activation capability. For a given experiment, GFP samples (in duplicate) of 1.5 mL of GFP (20,000 platelets/µL) were placed in the smaller Petri dishes and then secured to the vibration face of the VAED (contact experiments) or placed on a base 1 cm. below the suspended speaker (non-contact experiments). For all experiments, resting, non-exposed GFP, and GFP exposed to sonication served as non-exposure and max activation controls, respectively. All experiments were conducted over 40 min in a humidified incubator at 37 °C. For all sample conditions, serial aliquots of GFP were collected at 10-min intervals and analyzed via the PAS assay.

### 2.7. Effect of Epinephrine on Platelets Pre-Exposed to Snore

Gel-filtered platelets in small Petri dishes were exposed to snore-associated vibration (whole snore) as above for 40 min, with unexposed GFP in small Petri dishes serving as control. Following 10 min of exposure or non-exposure, 10 mM epinephrine (epi) was added to samples of both groups, i.e., vibration-exposed and non-exposed platelets. As a control for the epinephrine group, HEPES-modified Tyrode’s buffer was added to a set of exposed and no-exposed GFP in Petri dishes as well. All samples were then incubated at 37 °C for 40 min, and serial aliquots were removed at 10-minute intervals and analyzed via the PAS assay.

### 2.8. Effect of Hypoxia on Platelets Exposed to Snore

GFP was incubated for 30 min at 37 °C with overflowing CO_2_ in an incubator to induce a hypoxic state. To maintain hypoxia throughout the vibration experiment, the VAED was placed in a chamber that was pumped with 100% CO_2_. The pressure inside the chamber was kept at less than 2 psi. After 30 min of pre-incubation, the VAED was turned on, and GFP was exposed to snore-associated vibration (whole snore recording) at 100% acoustic intensity for 40 min as outlined above. Samples were collected at serial time points, and platelet activation was assessed via the PAS assay.

### 2.9. Effect of Aspirin on Snore-Mediated Platelet Activation

Aspirin (ASA, Sigma, St. Louis, MO, USA) was dissolved in sodium bicarbonate solution (324 mg ASA, 965 mg citric acid, and 1744 mg sodium hydrogen carbonate in 50 mL double-distilled H_2_O) and diluted to 125 μM final concentration. To verify the inhibitory efficacy of the ASA preparation, GFP was treated with ASA at a final concentration of 25 μM for 10 min at 37 °C. ASA-treated GFP was then exposed to 25 μM arachidonic acid (AA), with ASA-treated GFP and untreated GFP (no ASA, no AA) serving as control. All samples were incubated for 10 min at 37 °C, and the platelet activation state was determined. Separately, ASA-treated GFP, 37 °C × 10 min) was exposed to snore-associated vibration at 100% acoustic intensity for 40 min. as outlined above, with a non-vibration group as controls. In addition, GFP-only samples, GFP treated with epinephrine, and GFP exposed to hypoxia were prepared as outlined above. These specimens were then incubated with ASA at a final concentration of 25 μM at 37 °C for 10 min and similarly exposed to snore-associated vibration (whole snore recording) on the VAED over a 40 min period, with non-exposed samples as control. Samples were collected from all groups at serial time points, and platelet activation was assessed via the PAS assay.

### 2.10. Optoacoustic Vibration Patterns Generated by Snore Frequency Components

The VAED was utilized to obtain the fluid vibration patterns generated by snore frequencies. Smaller Petri dishes were secured onto the larger Petri dish vibration surface of the VAED with the cover off to allow contained fluid visualization. Water (3 mL) was placed in the secured smaller dishes, and the VAED was activated with defined frequencies at 100% output—as utilized for the activation experiments above, for 10 s as a high-resolution camera phone (4K@30/60 fps, 1080p@30/60/120/240 fps; gyro-EIS, OIS, Pixel 6 pro, Google, Mountain View, CA, USA) recorded the vibrational patterns. Frames of the videos generated were assembled as a composite for comparison.

### 2.11. Statistical Analysis

For all experiments, a two-tailed Welch’s *t*-test was used to compare the means of non-exposed to exposed platelets, with *p* < 0.05 indicating a significant difference.

## 3. Results

### 3.1. Mechanical Characterization of Vibro-Acoustic Exposure Device

Snoring was recorded in the sleep laboratory of the University of Arizona from OSA patients with a high-fidelity audio system. A typical snore recording (amplitude vs. time) is shown in Figure 2a for 14 s, normalized for depiction over a range of a −1 to 1 scale (AU, arbitrary units). A wide frequency range content was detected for recorded snoring, ranging from 100 to 1300 Hz (Figure 2b). The snore recording could be readily amplified and played back as an input signal for the actuation of the VAED. The characterization of the amplitude of the vibro-acoustic wave emitted at different levels of amplification by a laser vibrometer is shown in Figure 2b. The frequencies from 200 to 300 Hz and 850 to 950 Hz displayed a higher intensity and decibel content. Powering the VAED over a range of amplification from 0–100% in 25% increments revealed a range of emitted intensities (Figure 2c). This enabled quantification of the intensity of the reproduced snore and its induced vibration as a function of amplified level (volume).

### 3.2. Effect of Contact versus Noncontact on Snore-Mediated Platelet Activation

**Figure 3:**
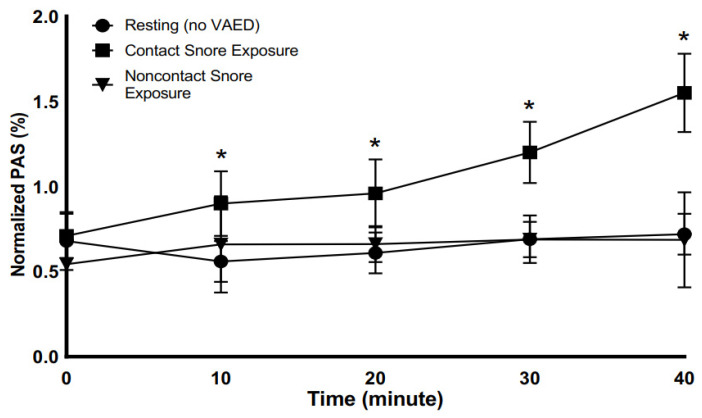
Platelet activity state (PAS) in response to whole snore exposure via the vibro-acoustic exposure device. Snore-exposed platelets increased activation with time of exposure, with significant difference versus non-contact and resting platelets detectable starting at 10 min of exposure (* *p* < 0.05).

Exposure of platelets to snoring, either as a composite recording (whole snore) or for the selected frequencies tested, resulted in platelet activation only in the contact mode, i.e., only for platelets subjected to direct transmission of vibration to the fluid platelet sample (Figure 3). Platelets exposed to identical snore frequencies and intensities in a noncontact mode, with an interposed air gap with no detectable fluid vibration, did not demonstrate activation over the tested 40-min period. There was no difference in platelet activation for the non-contact exposure group from that of a baseline resting control over 40 min as well. As a decision outcome of this first study, all subsequent experiments herein were conducted in contact mode.

### 3.3. Effect of Time of Exposure on Snore-Mediated Platelet Activation

In the contact VAED mode, platelets were exposed to 42.10 dB, i.e., 100% maximum intensity of snore, for 40 min. Snore exposure was compared to non-snore-exposed resting platelets. After 10 min of snore vibration exposure, a significant difference was already detectable versus resting platelets (Figure 3). Over the entire duration of the study exposure period, the snore-exposed samples were found to have a higher net platelet activity state than the resting sample at 20 min, with values of 0.96% and 0.61%, respectively (*p*-value = 0.003) and at 30 min with values at 1.20% and 0.69% (*p*-value = 0.001), respectively. The 40-min time point for snore-exposed samples had the highest increase in platelet activity state versus other time points and continued to be significantly different from the resting non-exposed samples with values of 1.55% and 0.72% (*p*-value = 0.001), respectively.

### 3.4. Effect of Snore Vibro-Acoustic Exposure on Platelet Morphology

**Figure 4:**
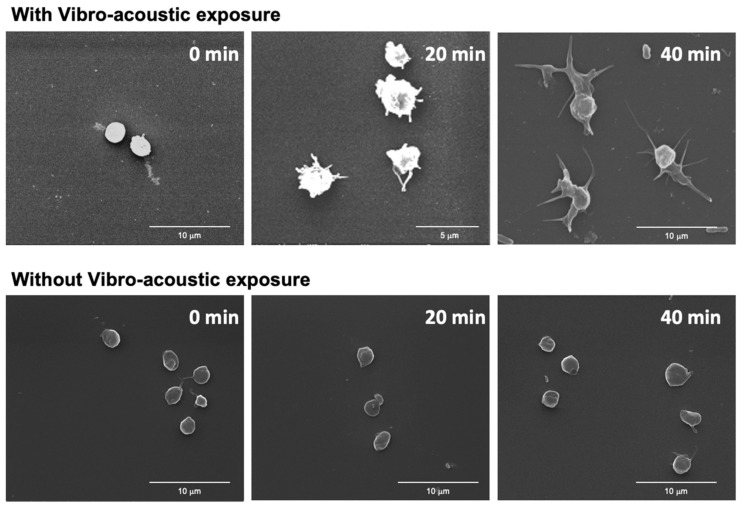
Effect of snore-associated vibration on platelet morphology. Top row—SEM Images of platelets exposed to whole snore-associated vibration for 0, 20, and 40 min. Bottom row—SEM of control platelets without snore-associated vibration exposure at 0, 20, and 40 min.

Continued exposure of platelets to snore-associated acoustic vibration led to progressive changes in platelet morphology (Figure 4). Notably, with time, an increase in the degree of platelet activation was detectable morphologically. In the absence of vibro-acoustic exposure, platelets were noted to retain an unactivated discoid morphology. In contrast, following 20 min of snore vibration exposure, platelets began to extend pseudopods. At 40 min, more pronounced pseudopod extensions were noted along with platelet spreading, indicative of greater activation.

### 3.5. Effect of Varying Snore Intensity on Platelet Activation

**Figure 5:**
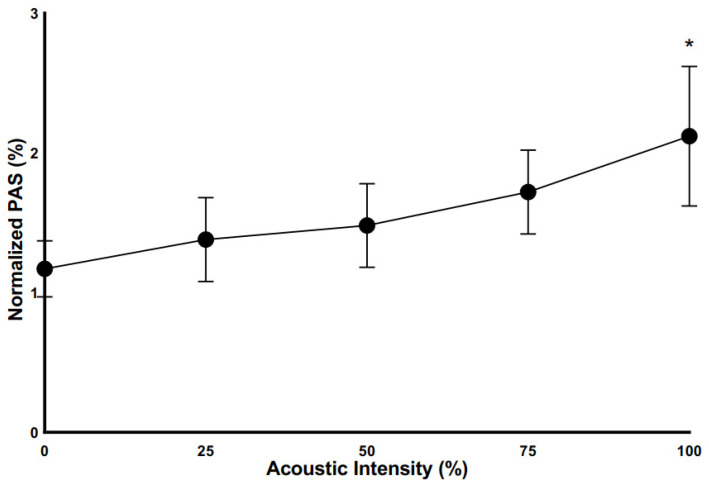
Platelet activation as a function of the intensity of vibro-acoustic exposure. All exposures for 30 min. (100% exposure, * *p* < 0.05 vs. non-exposure control).

Exposure of platelets to varying intensities of snore-associated vibration (whole snore recording) revealed varying degrees of activation. Over 30 min, a linear, progressive increase in platelet activation was noted, correlating with increasing intensity of vibration exposure (Figure 5). Progressing from 0 to 100% exposure in 25% increments resulted in an increase of activation from 1.2%, to 1.4%, to 1.5%, to 1.7%, and 2.1%, respectively. The increase observed at 100% was significant in comparison to the baseline 0% control (*p* = 0.047).

### 3.6. Effect of Differing Dominant Frequencies on Snore-Mediated Platelet Activation

**Figure 6:**
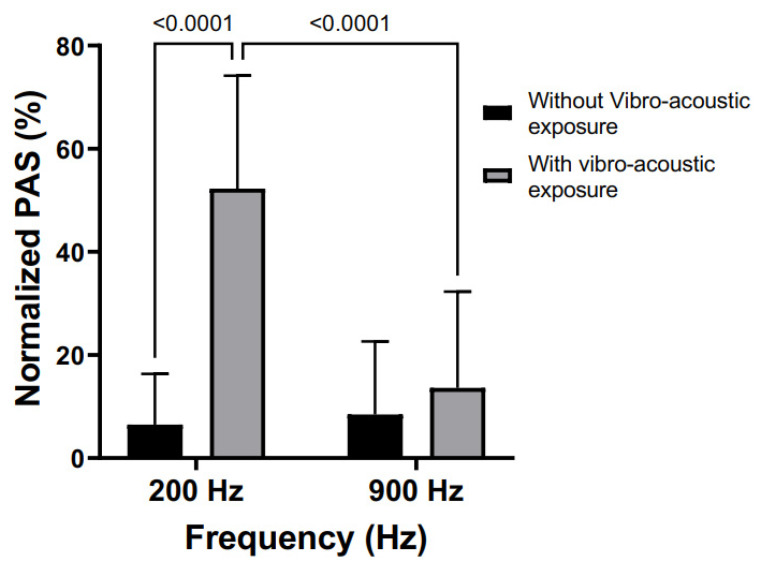
Effect of defined frequency exposure on platelet activation. All samples were exposed to continuous (40 min) frequency-mediated vibration via the VAED. (200 Hz and 900 Hz tested vs. non-vibration control).

Frequency spectrum analysis of snore recordings identified two high-energy frequency ranges: 150 to 250 Hz and 850 to 950 Hz (Figure 2). The median frequencies from the two ranges, i.e., 200 and 900 Hz, were used as representative of the dominant frequencies in snoring to determine the effect of frequency on platelet activity. The two frequencies were used at the same vibro-acoustic intensity corresponding to the amplified level of the 42.10 dB snore. Platelet activation state relative to the tested frequencies is shown in Figure 6. The frequency of 200 Hz had a greater effect on increasing platelet activation state than 900 Hz, achieving 53.05% versus 22.08%, respectively (*p* = 0.001). The controls for both were 15.92% and 13.78%, respectively.

### 3.7. Effect of Epinephrine versus Epinephrine + Snore-Associated Vibration on Platelet Activation

**Figure 7:**
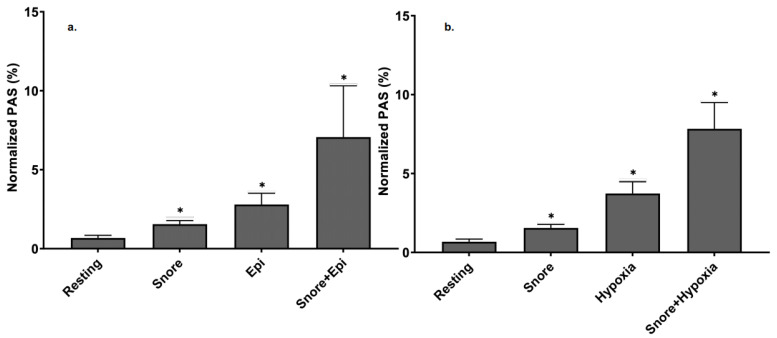
Comparative versus the additive effect of epinephrine and hypoxia with snore-associated vibration-mediated platelet activation. (**a**) snore exposure vs. epinephrine vs. combination of snore + epinephrine. (**b**) snore vs. hypoxia vs. combination of snore + hypoxia. (All agonists were significantly different from resting, * *p* <0.05).

Epinephrine alone was observed to activate platelets (Figure 7a). In comparison to snore vibration, epinephrine led to a platelet activity state of 2.79% ± 0.02, which was significant compared to 0.68% ± 0.01 for untreated control (*p*-value = 0.001). In comparison, snore vibration alone led to a PAS of 1.55% ± 0.01, which was also significant relative to the control (*p* = 0.001). Notably, the combination of epinephrine + snore vibration led to a much greater increase in platelet activation (7.06% + 0.08) than either epinephrine or snore alone (*p* = 0.001). The overall increase was > 10-fold relative to control, 2.5-fold > epi alone, and 4.5-fold > snore alone.

### 3.8. Effect of Hypoxia versus Hypoxia + Snore-Associated Vibration on Platelet Activation

Platelet exposure to hypoxia, under the conditions employed, resulted in platelet activation (Figure 7b). In comparison to snore vibration, hypoxia led to a PAS of 3.73% ±0.03, which was significant compared to 0.68% ±0.01 for untreated control (*p*-value = 0.001). In comparison, snore vibration alone led to a PAS of 1.55% ± 0.01, which was also significant relative to the control (*p* = 0.001). Like epinephrine, the combination of hypoxia + snore-associated vibration led to a much greater increase in platelet activation state, i.e., 7.83% ±0.08, than either hypoxia or snore alone (*p* = 0.001).

### 3.9. Combined Effects of Epinephrine and Hypoxia with Snore Vibration Platelet Activation

**Figure 8:**
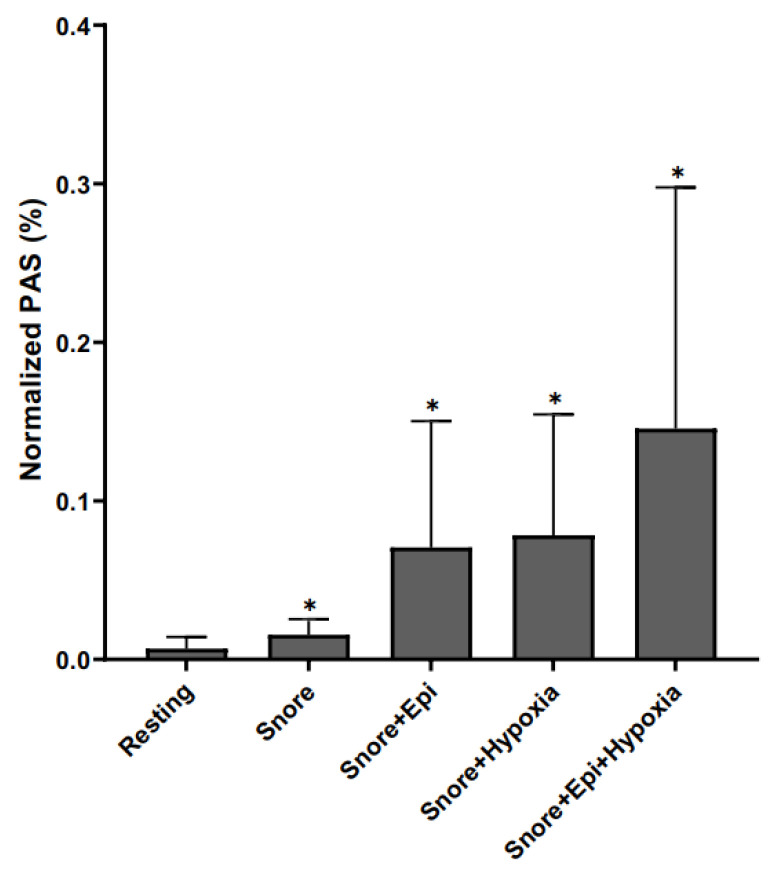
Additive/Synergistic effects of exogenous agonists. Snore vs. snore + epi vs. snore + hypoxia vs. combination of snore + epi + hypoxia. (All combinations of agonists are significantly different from resting, * *p* < 0.05).

The combination of snore-associated vibration with epinephrine and hypoxia led to an even greater degree of platelet activation than either epinephrine or hypoxia alone (Figure 8). Combining all three agonists led to a net synergy of activation. Compared to baseline control activation of 0.68%, the triple combination led to a net 14.55% ± 0.11. activation versus 7.06% for snore + epinephrine and 7.83% for snore + hypoxia.

### 3.10. Effect of Aspirin on Snore and Combinational Agonist-Mediated Platelet Activation

**Figure 9:**
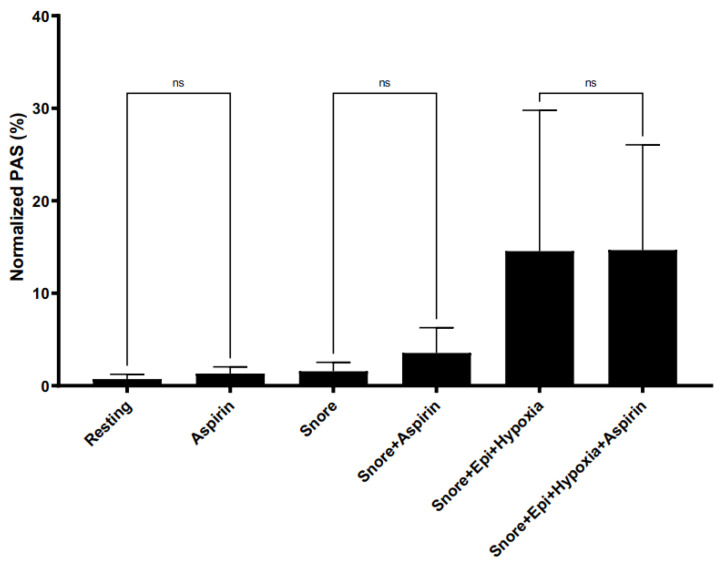
Effect of aspirin as a means of limiting snoring and combinational agonist-mediated platelet activation. Aspirin had no effect in limiting activation for each experimental condition versus its non-aspirin control).

Aspirin did not limit snore vibration-mediated platelet activation (Figure 9). The concentration of aspirin tested was observed to have no inhibitory effect on snore vibration-mediated platelet activation (Figure 9). In fact, a minor degree of activation was noted for aspirin-treated platelets exposed to snore vibration (25 μM), i.e., 3.54% versus 0.68% for snore alone. Aspirin had no effect in limiting platelet activation resulting from synergistic activation induced by snore vibration + epinephrine + hypoxia. As an overall control for this series of experiments, the aspirin preparation tested was found to be inhibitory of arachidonic acid-mediated platelet activation, with arachidonic acid resulting in 13.73% activation versus ASA + AA in 3.71%, *p* = 0.036.

### 3.11. Optoacoustic Vibration Patterns Generated by Snore Frequency Components

**Figure 10:**
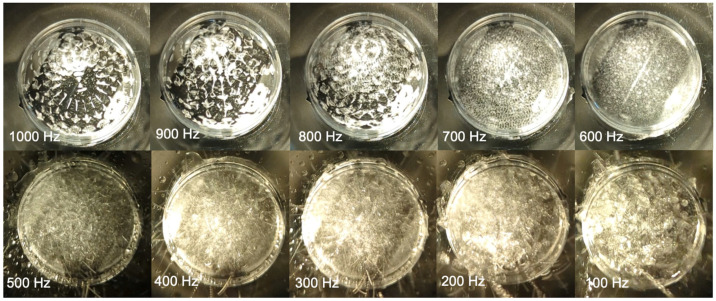
Vibro-acoustic effect of acoustic frequency on fluid dispersion and ejection. (1000–100 Hz).

A progressive degree of fluid disturbance and vibration was visually detectable and found to be inversely correlated with the frequency of exposure (Figure 10). At higher frequencies, shimmering optoacoustic patterns were noted, with limited disturbance to bulk fluid. As frequency decreased, increasing disturbance with fluid movement was detectable, with fluid ejection and splash observed at <500 Hz. Low-frequency bandwidths induced noticeably greater fluid oscillation than high-frequency bandwidth exposure.

## 4. Discussion

Obstructive sleep apnea is a highly prevalent disease and is associated with significant cardiovascular morbidity and mortality. Of particular concern is the association of OSA with stroke. Relatedly, snoring alone has also been found to result in an increased risk of stroke [11,12,15,19]. Central to stroke pathophysiology are thrombosis and thromboembolic phenomena, all based on and initiating with platelet activation [12,19]. In the present study, we determined that the vibrational component of snoring, common to both intense snoring and snoring associated with OSA, is a direct “mechanical” agonist of platelets, resulting in platelet activation. Vibration-mediated activation was found to be associated with exposure time and intensity, i.e., dose-dependent, with low-frequency components being the most activating. Furthermore, our results suggest that snore-associated vibration may act synergistically with both catecholamines and hypoxia—both biochemical responses associated with OSA—to induce an even greater level of platelet activation than either agonist alone. Aspirin, a classical antiplatelet agent, was found to have no efficacy in limiting snore vibration-mediated platelet activation alone or in the setting of synergistic epinephrine and/or hypoxia agonists.

### 4.1. Vibro-Acoustic Stimulation of Biological Cells

Sound applied to an aqueous fluid is a pressure wave propagating within the medium at speeds on the order of 1500 m/s. At the frequency of audible sound, such as that associated with snoring, the wavelength is on the order of several meters. Any microscale heterogeneity, such as platelets in the fluid, will essentially experience a constant pressure field, leading to negligible pressure forces. As such, the absence of a pressure gradient or any other sudden change in the corresponding force field experienced by the platelet will not result in activation. Platelet activation requires forces acting on length scales on the order of the platelet diameter (2–4 µm). Long-wavelength audible acoustic waves cannot produce so-called acoustic cavities that may be able to concentrate mechanical energy on a scale smaller than the wavelength. Furthermore, low-intensity snore acoustic waves cannot form cavitation, which is a known activator of cells [48].

The loss of acoustic momentum that results from attenuation or dissipation of the sound field in a viscous fluid may result in a nonlinear time-independent fluid motion, i.e., stationary vortices, known as acoustic streaming [36,37,49]. The scale of any acoustic streaming happens on the scale of the wavelength, therefore eliminating that mechanism as a possible activator of platelets.

In addition to acoustic waves, we have considered the effects of vibration on platelet activation. As shown in Figure 10, the vibrations of the fluid receptacle create ripples on the surface of the fluid, which are known as Faraday waves [49]. Faraday waves are nonlinear standing waves occurring when the vibration frequency exceeds a critical value, the fluid surface becomes unstable, and protrusions/ripples form on the surface. The amplitude of the Faraday waves scales to the intensity of the vibro-acoustic exposure device. Fluid flow associated with gradients in velocity resulting from these instabilities may potentially lead to shear forces on microscale platelets. Our study demonstrates that exposure to snoring via a contact configuration of the VAED led to platelet activation via vibration as opposed to audible acoustic waves. Disturbed fluid motion, with associated non-physiological shear forces, is a known activator of platelets [37,50,51,52,53]. With the VAED operating in the contact configuration, stronger mechanical vibrations and vibro-acoustic waves are likely to be operative on platelets versus acoustic waves alone. Furthermore, with a significant impedance mismatch between air and sample fluid in the noncontact mode of operation, one expects that platelets will be subjected to primarily the effect of the low-intensity vibro-acoustic waves, with air inducing greater damping of high versus low-frequency vibro-acoustic waves [54,55,56]. This is consistent with the absence of platelet activation observed for the noncontact studies.

### 4.2. Vibration Effects on Stresses Acting on Flowing Platelets

Vibration-related shear has been described as an activator of flowing cells [44]. OSA and snoring vibrations that are transmitted to adjoining carotid arteries and jugular veins induce flow disturbances via fluid–structure interaction that is transmitted to the vessel walls, where these vibrations are translated into random flow disturbances characterized by instantaneous acceleration forces acting on the flowing blood. Out-of-phase accelerations acting on a cell nucleus or its intracellular constituents appear to play a role in the cellular response to vibrations [44]. High-frequency flow disturbances such as those induced by OSA and snoring may generate both laminar viscous shear and turbulent stresses. The latter, known as Reynolds stresses, are random and can easily increase the total stresses acting on flowing platelets by an order of magnitude [57,58]. Platelets respond and activate earlier by such a combination of stresses. The turbulent Reynolds stresses contain high-frequency random vibrations that act directly on the platelet membrane and its receptors. Those are then transduced to the intracellular components via the cytoskeleton and cytoplasm of the platelet and may initiate earlier activation [34,59,60].

### 4.3. Effects of Vibro-Acoustic Dose on Platelet Activation

Our studies demonstrate that with increasing length of snore-associated exposure, greater platelet activation occurs (Figure 3). This concept of dose, i.e., intensity × time, is well established for the mechanical activation of platelets [45,61,62]. In the case of shear stress, this phenomenon is referred to as net stress accumulation. In prior work, our group has shown that for a given intensity of stress, the degree of platelet activation increases with the repetitiveness (frequency) of stress exposure [63,64]. Furthermore, even with cessation of exposure, platelets are sensitized and will continue to activate proportionally to the magnitude of the initial shear stress dose [45]. This progressive increase in platelet activation is seen here as well with snore-associated acoustic vibration exposure.

The importance of time of exposure, dose, and post-stress sensitization is reflected in our studies examining whole snore exposure versus selective frequency exposure. For whole composite snore exposure, a PAS of 1.55% was achieved at 40 min. By contrast, at 200 Hz, a PAS of 53.05%, and at 900 Hz, a PAS of 22.08% was achieved. This difference in activation may be explained by understanding the actual period of vibration exposure induced with each snore regime. For whole snore—i.e., actual snoring—sound and vibration events only occur intermittently and episodically. In considering a series of repetitive snores as in actual snoring, the duty cycle of sound exposure represents 20–25% of the time interval between snoring events, with 70–80% of the time being silent, free of vibrational and mechanical exposure. By contrast, for the single-frequency studies, sound and vibration were produced continuously without interruption, leading to 40 min of intense mechanical exposure. This longer duration of persistent vibration is reflected in the greater degree of platelet activation observed.

At 42.10 dB for 40 min, platelets are adequately stimulated to activate. For reference, 42.10 dB corresponds to a pressure of 0.025 dynes/cm^2^ [40,64]. Obstructive sleep apnea is designated as moderate or severe when the snoring episode has a duration of 30 min or more [14]. It has been suggested that exposure to a high vibration intensity over a short period of time may activate platelets in a way similar to that of a lower intensity over a longer time [65]. This may explain the possibility of significant platelet activation even at low vibration intensities if exposure exceeds 40 min.

### 4.4. Effect of Frequency on Platelet Activation

Examination of the frequency spectrum of typical snore recordings revealed two high-energy content regions centering around 200 Hz and 900 Hz. Of these two frequencies, 200 Hz led to greater platelet activation compared with 900 Hz, despite exposure to the same (100%) intensity of vibration. These results suggest that the low range frequency leads to greater oscillation and fluid disturbance, directly imparting activating forces on the platelets. This is consistent with the visibly detectable fluid disturbances observed (Figure 10). We have previously shown that flowing platelets are sensitive to specific frequencies in their shear stress trajectories through gaps in prosthetic heart valves, with higher thrombin generation observed in the 82–94 Hz band despite a lower intensity, 0.56 dB, than other frequency regions [66]. Furthermore, short exposures of platelets to low oscillatory frequencies trigger significant thrombin generation compared to constant low-shear stress, and thrombin generation continues to increase even after cessation of oscillatory flow [58,64]. This suggests a differential stress-biochemical coupling effect, where the frequency components are sensed and mechano-transduced by the platelets, triggering downstream biochemical events, e.g., receptor activation, cytoskeletal changes, and granule release. This differential stress-biochemical coupling mechanism is observed in other cell types, including vascular endothelial cells, fibroblasts, and bone mesenchymal stem cells [67,68,69].

The midrange speaker utilized in these studies has an operating frequency range of 100 to 1500 Hz, which cannot explain, based on mechanical intensity response to voltage stimulus, the difference in activation for the two tested frequencies. The variation in activation as a function of frequency is likely due to the difference in response of the fluid and contained platelets to vibrations generated by these defined frequencies.

### 4.5. Combined Effects of Vibration and Biochemical Agonists on Platelet Activation

Patients suffering from severe OSA tend to have intermittent spikes in blood catecholamine levels associated with startle and awakening after intermittent apneic episodes. Similarly, these patients have transient episodes of relative hypoxemia [11,12,15,16,21]. Our results demonstrate and confirm that both epinephrine exposure and hypoxia induce platelet activation. Notably, our results extend insight into potential mechanisms operative in OSA. The finding that snore-associated vibration activates platelets and that this activation is further amplified in the setting of epinephrine and hypoxia, or conversely that snore-associated vibration amplifies the basal activation observed with these known platelet agonists, is suggestive of additional mechanisms that may be operative and underlie the demonstrated increased thrombosis and stroke risk of OSA and loud snoring. Furthermore, these mechanisms may also contribute to an overall increased cardiovascular risk [11,12,15,16,19,21]. Underlying this synergy of agonists may be alteration of platelet membrane porogenicity induced via snore-associated vibration, enhanced exposure of agonist receptors resulting as a result of vibrational change, or facilitated presentation and mixing of biochemical agonists, or enhanced activation initiation via hypoxia-mediated mitochondrial and internal energy changes. These potential mechanisms underlying the observed synergies are plausible, though they remain to be experimentally defined.

### 4.6. Combined Effect of Aspirin, Agonist, and Snore-Associated Vibration on Platelet Activation

Aspirin is the prototypic antiplatelet agent, demonstrated to reduce prothrombotic cardiovascular risk [70]. It is well established that the mode of action of aspirin is via inhibition of arachidonate and thromboxane A2 pathways within the platelet [71,72]. Over the past few years, it has been demonstrated that ASA has had limited efficacy clinically in limiting platelet activation in settings of elevated shear stress and flow disturbances, e.g., as in the setting of implanted mechanical circulatory support devices, in fact, driving excessive bleeding [73]. Furthermore, definitive studies have recently established that ASA has a limited ability to inhibit shear-mediated platelet activation [74,75,76]. Underlying this lack of effect has been the realization that shear-mediated platelet activation does not involve the classical biochemical agonist receptor pathways but rather mechanisms such as mechano-transduction, mechano-destruction with membrane damage, and mechanosensitive channel activation [34,77]. It is very likely that the observed lack of efficacy of ASA in the setting of snore vibration-mediated platelet activation relates to this similar dichotomy as to the pathway of activation involved, with mechanical means differing from traditional biochemical agonist pathways. Consistent with this have been clinical studies demonstrating the limited efficacy of aspirin in reducing OSA-associated thrombosis and stroke [78,79].

Beyond the lack of inhibitory efficacy of aspirin, we observed a slight trend toward increased platelet activation for aspirin-treated platelets exposed to snoring. Although we caution that these data are limited and in vitro, it has been reported in other settings for aspirin to have the potential for a paradoxical prothrombotic effect [80], with conceivable mechanisms related to variable Cox-1 vs. Cox-2 reactivity [81,82] or variable platelet subpopulation reactivity [83], among other possibilities.

## 5. Limitations

The vibro-acoustic exposure device, constructed from plastic and fiber materials, has material properties that differ from those of the airway, neck, and blood vessel tissue in patients. As such, the vibration resonance, wave transmission, and fluid–structure interactions may differ from the in vitro testing here versus the in vivo patient situation. The present study utilizes sound recordings typical of patients with OSA. Despite this, patients over a range of body habitus, differing age, and degree of throat and neck tissue laxity may have differing frequencies of snoring and differing coupling characteristics regarding fluid–structure interactions and transmission of sound-mediated vibration to blood and fluid and contained platelets. The finding here may represent potential mechanisms operative for a particular defined spectrum of individuals. The study here utilized platelets in a fixed dish. Future studies are planned for snore vibration exposure to flowing platelet samples.

## 6. Conclusions

Obstructive sleep apnea and loud snoring both carry an increased risk of stroke. Thrombotic stroke in these conditions has traditionally been hypothesized to be driven by biochemical and inflammatory mediators. In the present study, we demonstrate that snore-associated vibration is a mechanical agonist of platelets that leads to platelet activation. Snore-associated vibration activates platelets in a dose-dependent fashion, with activation continuing to increase with increasing time of exposure. Low frequency, 200 Hz, appears to activate platelets greater than higher frequencies. Furthermore, snore-associated vibration as an agonist of platelet activation is synergistic with known activators and risk factors of thrombosis in OSA and snoring, i.e., catecholamines and hypoxemia. Aspirin, a standard clinical antiplatelet agent, is ineffective in limiting snore vibration-mediated platelet activation. These studies identify a new risk factor and potential mechanism operative in snoring and OSA, i.e., acoustic vibration-mediated platelet activation. Further defining the mechanical-cellular coupling mechanisms operative will afford opportunities for translational advancement to reduce the clinically significant consequences associated with thrombotic and thromboembolic consequences of OSA and snoring.

## Data Availability

Data that support the findings of this study are available for the corresponding author upon request.
